# An Overview of Alkylresorcinols Biological Properties and Effects

**DOI:** 10.1155/2022/4667607

**Published:** 2022-01-05

**Authors:** Anastasia A. Zabolotneva, Olga P. Shatova, Anastasia A. Sadova, Aleksandr V. Shestopalov, Sergei A. Roumiantsev

**Affiliations:** ^1^N. I. Pirogov Russian National Research Medical University, 1 Ostrovitianov str., 117997 Moscow, Russia; ^2^Center for Digital and Translational Biomedicine, Center for Molecular Health, 32 Nakhimovskiy Prospekt, 117218 Moscow, Russia

## Abstract

The investigation of alkylresorcinols has drawn an increasing interest recently. Alkylresorcinols (ARs) are natural chemical compounds synthesized by bacteria, fungi, sponges, and higher plants, possessing a lipophilic polyphenol structures and a myriad of biological properties. Human takes ARs as a component of a whole grain diet (from whole grain rye, wheat, and barley products), and thus, alkylresorcinols are frequently used as whole grain intake markers. Besides, ARs are considered as promising bioregulators of metabolic and immune processes, as well as adjuvant therapeutic agents for antimicrobial and anticancer treatment. In this review, we attempted to systematize the accumulated information concerning ARs origin, metabolism, biological properties, and their effect on human health.

## 1. Introduction

The interest in the investigation of the biologically active components of grain products has been progressively rising for the past few years due to the intriguing recent observations that a whole grain diet is strongly correlated with the lower risk of obesity, diabetes, and some cancers, namely, breast, colon, and prostate cancer [1] and that a gluten-free diet is inversely associated with type 2 diabetes development [2]. Besides, in the last decade it has become clear that not only diet, but also the human gut microbiome provide a lot of regulatory molecules that influence host metabolism and immunity functioning directly or indirectly [3] through the formation of interconnections between the gut and adipose tissue [4], the gut and nervous system [5], the gut and immune system [6], etc. In this regard, the investigation of alkylresorcinols is of particular interest.

**Figure 1:**
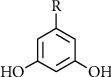
General structure of ARs. R-alkyl or alkenyl side chain, oxygenated R is also possible. The R position may be changed and presence of additional polyphenolic rings is possible depending on the type of organism synthesizing ARs.

ARs are naturally synthesized by plants (like rye, wheat, and barley), fungi, and bacteria and possess lipophilic polyphenol structures (Figure 1) along with many biological activities. Type III polyketide synthases (PKSs) found in all domains of life are in charge of the synthesis of AR as well as other aromatic polyketides [7]. Interestingly, *srs*-like operon in prokaryotes, which contains *srsA* gene (Streptomyces resorcinol synthase A) encoding PKS type III, is commonly found among Gram-positive and Gram-negative bacteria (including Actinobacteria, Cyanobacteria, Proteobacteria, and Halobacteria) [8]. This fact indicates that phenolic lipids are widely distributed among the organisms of this domain of life. In bacteria, ARs are the components of plasma membranes, which may increase their rigidity and thus protect cells from dangerous compounds like antibiotics or from unfavorable environmental conditions. However, the actual functions of ARs found in different organisms remain to be elucidated and are currently under investigation. Herein, we aimed to observe ARs biological properties known to date and to review their effects on human metabolism in link with chronic disorders.

## 2. The Structure and Synthesis of ARs

As mentioned above, ARs are lipophilic molecules with polyphenol structure (Figure 1), which exhibit various biological functions and as many different aromatic polyketides are synthesized by type III PKSs [7].

**Figure 2:**
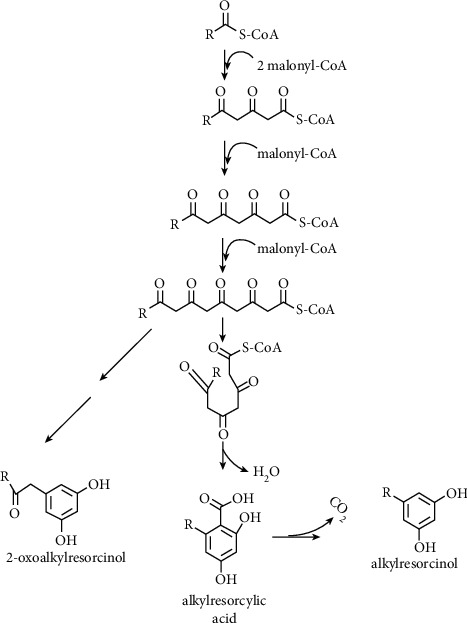
A proposed biosynthetic pathway of ARs by PKSIII according to [10].

In the reactions catalyzed by these enzymes, a stater substrate such as acyl-CoA and an extender substrate such as malonyl-CoA are iteratively decarboxylated. Following the condensation reaction, the resultant polyketide chain is then cyclized to yield dissimilar bioactive natural compounds dramatically diverse in their structure. This structural variety of polyketide scaffolds is governed mainly by the enzyme's selectivity to starter and extender substrates, the number of condensation reactions, and the mode of ring closure of the resultant polyketide chains [9] (Figure 2).

## 3. The Natural Sources of ARs and Their Absorption

On the first glance, it seems that the only source of ARs in the human diet is the outer layers of wheat and rye kernels where they are present in significant amounts [11]. At the same time, ARs were found in mango peels, leaves, and seeds [12]. Besides, alkylresorcinols were shown to be one of the main components in whole grain products possessing marked antiproliferative properties against the growth of cancer cells in humans [13]. Additionally, ARs may enter the human body as preservative agents (particularly, 4-hexylresorcinol) found not only in food, but also in several medications, toothpaste, and cosmetics [14]. Interestingly, the human gut microbiome can be considered as a source of ARs as well. Prokaryotes (namely, Actinobacteria, alpha-, delta-, and gamma-Proteobacteria, and Cyanobacteria) are able to synthesize different monoalkylresorcinols [15–18] which demonstrate anticancer, antifungal, anti-inflammatory, antimicrobial, antiparasitics, antioxidant, and genotoxic activities [19]. It is worth to note that, in the study of Martins et al., a phylogenetic analysis of the genes coding for type III PKS enzymes revealed a wide abundance of such sequences among different members of Cyanobacteria phylum [18]. In addition, the presence of *srs*-like operon structures coding for type III PKS in numerous Gram-positive and Gram-negative bacterial species was reported in the study of Funabashi et al. [8]. Taken together, these data demonstrate that the ability to synthesize ARs is common to many prokaryotes. Thus, one should not exclude the bacterial origin of ARs found in human biological samples, although, to date, there is no unambiguous evidence that the human microbiome contributes to the body pool of ARs.

**Figure 3:**
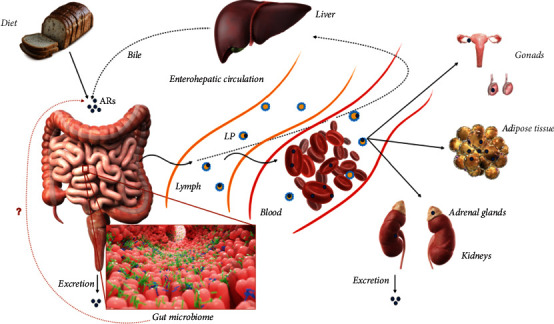
ARs absorption and metabolism. ARs enter the human body mainly with whole grain products, although gut microbiome origin of ARs is also possible. ARs are transported through the lymph and blood as a part of lipoproteins (LP) toward the liver and other tissues. ARs are metabolized in the liver and excreted through the kidneys with urine or through the intestine with feces.

Intriguingly, small concentrations of ARs, namely, 3,5-dihydroxybenzoic acid (DHBA) and 3-(3,5-dihydroxyphenyl)-propanoic acid (DHPPA), were found circulating in the blood of volunteers abstained from consuming the whole grain wheat and rye for one week [20]. The small concentrations of these compounds could arise from accidental consumption of cereal products containing minor amounts of ARs or liberation of ARs from stored body pools [21], or they could be produced by the gut microbiome. However, according to the authors, ARs with long side chains (C15-C25), that are likely to have plant origin, predominantly circulated in plasma (Figure 3).

Unfortunately, no researches evaluating the concentration of other types of ARs, including those produced by microbes, have been conducted so far.

The absorption of ARs is commonly investigated using an ileostomy model [22, 23]. The observations in pigs, rats, and humans demonstrate that the concentration of ARs in plasma rises proportionally to the intake of ARs as a food component, and similar results were shown for different model organisms [24]. At the same time, only 50–70% of the consumed ARs are absorbed [25]. The absorption of ARs occurs apparently in the small intestine by passive diffusion and may also happen similarly to tocols with the help of active transport provided by scavenger receptor class B (SR-BI) [26]. Next, enterocytes pack ARs into chylomicrons and release them to the lymphatic system. Another proposed way of ARs absorption concerns the direct transfer of these molecules to high-density lipoproteins in the intestine and their subsequent distribution into blood lipoproteins [27]. Thus, the major fraction of ARs in plasma is included in different lipoprotein (LP) fractions, namely, very low-density LP (VLDL), high-density LP (HDL) and low-density LP (LDL), while none of ARs are present in the water fraction of blood plasma. Indeed, VLDL and HDL are considered the main carriers of ARs in fasting samples (Figure 3).

Further pathways of ARs utilization in the body embrace their distribution to the membranes of red blood cells as well as their transport to hepatocytes and different cells and tissues. Interestingly, ARs may shorten the erythrocytes lifespan by destabilization of their membranes, and this haemolytic activity of ARs homologs depends on the degree of side chain unsaturation and is inversely proportional to the chain length [28].

Based on the data reported above, ARs and their metabolites, namely, DHBA and DHPPA, seem to be promising biomarkers of whole grain consumption [22]. Similarly, the rising of ARs levels in blood may serve as the evidence of the breach of a gluten-free diet. Finally, the study of ARs and their homologs will be of particular importance in the investigation of gluten effects in the wider population [29].

## 4. ARs Metabolism

**Figure 4:**
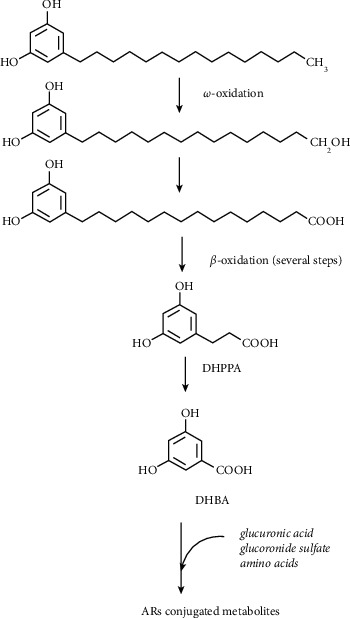
ARs metabolism in mammalian cells (pentadecylresorcinol (C15 : 0) is shown as an example). Hydroxyl groups on the phenolic ring are conjugated with amino acids, glucuronide, and/or sulfate groups during metabolism.

The metabolism of ARs in the liver consists of two phases and is similar to the oxidation of tocopherols. The first step results in the introduction of a hydroxyl group at the end of the alkyl tail of the molecule in the *ω*-oxidation reaction catalyzed by CYP4F2 (Figure 4). Next, the hydroxyl group is oxidized to a carboxylic acid followed by *β*-oxidation. ARs alkyl chain is shortened in this way resulting in two main metabolites, DHPPA and DHBA, which are water soluble molecules. During the second phase, the conjugation of the reaction products (ARs, their intermediates, and final metabolites) with glucuronide sulfate groups and amino acids happens, which significantly improve the urinary excretion of the molecules. Since the two AR metabolites, DHPPA and DHBA, can be detected in both human plasma and urine [30], they can be considered as biomarkers of whole grain wheat and rye intake [31] (Figure 4).

The application of radiolabeled ARs in the investigation of ARs metabolism in animals demonstrated that they are accumulated in the body in insignificant amounts [32]. However, in case of prolonged feeding with ARs, they are stockpiled in adipocytes and blood cells, as it was demonstrated in rats [23]. In addition, the body pool of ARs may include those molecules incorporated into the membranes of lipid transport vesicles or associated with plasma proteins [33].

## 5. The Biological Effects of ARs

ARs have been studied using *in vitro* experiments and meta-analysis, which have revealed a wide range of bioactivities exhibited by these molecules, namely, antimicrobial, anticancer, antilipidemic, antioxidant, and others described below.

### 5.1. ARs Are Autoregulators in Microbial Communities

In a population of bacteria, one can identify vegetative or actively proliferating cells and the dormant ones, which enter the quiescent state and do not divide. These dormant cells form metabolically inactive endospores, which are in charge of the establishment of persister cells in the given population. Endospores are characterized by high resistance to such unfavorable environmental conditions as radiation and high temperature, as well as by tolerance to antibiotics and antimicrobial chemicals [34]. Thus, the emergence of persister cells is one of the mechanisms of antibiotic resistance development leading to chronic bacterial infections and metabolic shifts in host organisms [35–37]. In this regard, it is of particular interest that ARs (namely, 4-hexylresorcinol) in combination with antibiotics were shown to dramatically decrease the number of germinating *B. cereus* spores both in liquid medium and on agar plates compared to the treatment with antibiotics alone [34]. These results are consistent with the data from other studies dedicated to the investigation of ARs autoregulation potential [38]. We propose that ARs entering the human body with food products should be considered as autoregulators in the gut microbial community, which may influence the regulatory axes «gut-immune system», «gut-nervous system», «gut-adipose tissue».

### 5.2. Antimicrobial Properties of ARs

Nowadays, clinical practice faces the problem of increasing antibiotic resistance of pathogenic bacteria. In an attempt to address this issue, two approaches have been developed, the first being the search for new powerful antimicrobial agents, and the other being the use of antibiotic adjuvants capable of enhancing the antimicrobial activity of the commonly used chemicals. For instance, Nikolaev et al. [34] tested the combination of ARs with 12 clinically used antibiotics against a broad range of pro- and eukaryotic pathogenic microorganisms. They found the application of 4-hexylresorcinol as an adjuvant to polymyxin to be highly efficient against the germination of bacterial dormant forms (spores) and to prevent the formation of antibiotic-resistant persister cells. The stunning results were obtained with the use of *an in vivo* mouse model of *K. pneumoniae*-induced sepsis: 75% of the animals recovered from the infection after treatment with the combination of ARs and the antibiotic, in contrast to the group receiving the antibiotic alone, in which none of the animals were cured. Thus, the authors conclude that ARs such as 4-hexylresorcinol can be used as an adjuvant to increase the efficiency of several known antibiotics and to decrease the risk of antibiotic resistance development.

ARs display antimicrobial activity by incorporation into the cell walls of microorganisms. Gram-positive bacteria possessing peptidoglycan-based outer cell wall were shown to be highly sensitive to ARs treatment. On the contrary, Gram-negative bacteria, with their additional outer lipid membrane, and yeast and other fungi with complex chitin and polysaccharide-based cell walls seem to resist ARs insertion into their membranes. Besides, the presence of lipid structures and mycolic acid makes the cell surface more hydrophobic, which is a strong obstacle for ARs penetration. According to the published data, the antimicrobial activity of n-alkylphenols is higher in those with longer alkyl chains, but the enrichment of microbial cell walls by lipids interferes with the ARs activity against microorganisms [19]. They suggest that the biological activity of ARs is likely governed by their ability to act as structural modifiers of biopolymers and supramolecular structures such as membranes [12, 19, 39, 40]. Presumably, ARs increase the membrane viscosity by association with lipid molecules and macromolecules within membranes, inhibiting their functional activities. Additionally, a dose-dependent interaction of ARs with DNA and proteins was reported, which is mediated by the formation of reactive oxygen species (ROS) and by the activation of RpoS (RNA polymerase, sigma factor S) in case of the increase in the concentration of ARs within the cell [41]. Based on these facts, it seems unlikely that ARs may cause microbial resistance, since their action is nonspecific and extends from bacteria to protozoans and helminths [19].

### 5.3. ARs Influences Gut Microbiome Composition

Gut microbiota not only closely interacts with dietary products, but is also interconnected with genetic factors, being one of the important causes of metabolic disorders and obesity development. However, the mechanisms underlying the influence of gut microbiota on host metabolism remain to be elucidated. Studies in animals and humans have demonstrated a strong correlation between the alteration of the microbial composition in the intestine, manifested in the shift toward increased energy harvest, and the obese phenotype [42, 43]. Additionally, the increasing ratios of Firmicutes (such as *C. coccoides*, *C. leptum*, and *Enterococcus*) to Bacteroidetes (such as Bacteroides and Prevotella) within the microbial community of the gut were reported to be associated with obesity and metabolic disorders [44]. As a whole grain diet was demonstrated to have prebiotic effects [45], the study in which the fecal microbiota in mice supplemented with ARs were examined is of particular interest [46]. Oishi et al. managed to show that food ARs significantly increased the amount of Prevotella and reduced the amount of *Enterococcus* in mice feces. Additionally, the fecal amount of the latter bacteria positively correlated with body weight and fecal bile acids but negatively correlated with the fecal amount of total lipids. It was suggested that the metabolic improvements demonstrated in this study were stimulated by ARs consumption, which thus manifested their prebiotic effects. These data are in concordance with the results of the investigation of antiobesity effects of prebiotic supplementation in humans and other animals [45]. It is also reported that polyphenols from beverages, fruits, and vegetables influence, i.e., may stimulate or prevent, the growth of bacteria in the gut [47].

On the other hand, in a randomized, controlled cross-over trial comprising two 8-week dietary intervention periods in 60 Danish adults exhibiting an increased metabolic risk profile, it was demonstrated that a whole grain diet, compared with a refined grain diet, led to weight loss and to the decrease of low-grade systemic inflammation markers without significantly changing the fecal microbial composition, diversity, or functional potential [24]. It is important to note that the effect of whole grains on inflammation markers was still evident after adjustment for weight loss. Interestingly, the concentration of the proinflammatory cytokine interleukin-6 (IL-6) in fasting serum was lowered in response to the intake of ARs C17 : 0, rye products being the main source of it [24].

However, the consumption of whole grains for 8 weeks did not affect much either glucose or lipid metabolism; liver fibrosis markers, metabolic satiety-regulating hormones, or blood pressure did not show any changes in the tested individuals as well compared with refined grain intake. Thus, one may speculate that the body weight of the trial participants supplemented with whole grain products was reduced mainly because the decreased energy intake for this type of food leads to satiation faster in comparison with refined grain products. Moreover, according to this study, the whole grain diet did not influence gut permeability [24].

At the same time, the fact that the amount of proinflammatory markers circulating in the blood is strongly inversely correlated with whole grain biomarkers, such as ARs, suggests a great potential of alkylresorcinols as health-promoting agents.

Contradictory results of the studies described above may result from different methods and approaches of microbiome analysis as well as from the diversity in metabolic characteristics of the investigated populations. Nevertheless, whole grains may be considered as a part of a healthy diet providing the normalization of body weight and reduction of systemic inflammation.

### 5.4. ARs Regulate Sirtuin (SIRT) Activity and Show Antiaging Potential

Restriction in calorie intake has been associated with the prolonged lifespan, and despite the fact that this phenomenon was confirmed in multiple epidemiological studies and different animal models, its underlying mechanisms have not yet been revealed [48]. To date, the observations associate the reduced glucose concentration in cell growth medium with the increased expression of sirtuins, particularly SIRT1 [49]. Sirtuins (SIRTs) belong to a class of proteins with NAD+-dependent deacetylase or adenosine diphosphate-ribosyltransferase activity and have been shown to participate in diverse cell functions such as DNA repair and recombination, gene silencing, senescence, and responses to stressors [50]. ARs were demonstrated to modulate the activity of SIRT1 by affecting the enzyme structure, most likely by allosteric activation of the enzyme, with no effect on the SIRT1 expression level [51]. According to this study, ARs are able to activate sirtuins in mammalian cells, as it was demonstrated that the percentage of histone acetylation in the human monocyte cell line THP-1 significantly decreased in response to C17 AR. Mediated by another SIRT family member, namely, Sir2, ARs were shown to prolong *D*. *melanogaster* lifespan in conditions of restricted calories intake [51]. Among other things, ARs addition to the 3T3-L1 cell culture prevented triglyceride accumulation in cells, indicating ARs potential to block triglyceride synthesis *in vivo* [52]. Numerous studies demonstrate that ARs alter the metabolism of lipids in a SIRT-mediated pathway, likely to forestall the development of metabolic syndrome [51].

### 5.5. ARs and Wound Healing

It has been known for decades that certain ARs possess antiseptic properties along with a broad-spectrum antimicrobial activity [53, 54]. Thus, 4-hexylresorcinol was demonstrated to significantly decrease the expression of tumor necrosis factor *α* (TNF-*α*) in RAW264.7 cells and in a rat burn wound model [55]. Concurrent with TNF-*α* suppression, 4-hexylresorcinol also induced rapid epithelization and collagen regeneration in animals, compared to the control group. These beneficial properties of hexylresorcinol have been successfully applied in many cosmetics products with antiaging and lightening effects [56, 57].

### 5.6. ARs May Prevent Muscle Atrophy

A decline in muscle mass, termed muscle atrophy, occurs under conditions of muscle disuse (e.g., immobilization, denervation, and muscle unloading), fasting, and aging and accompanies such diseases as cancer cachexia, Cushing's syndrome, sepsis, diabetes mellitus, and chronic renal failure [58]. From the point of view of biochemistry, muscle atrophy can be caused by decreased protein synthesis and/or increased protein decay [59]. Aging- and inactivity-mediated loss of muscle strength is associated with the accumulation of intramuscular triglycerides with the simultaneous progressive decrease in the mass of muscle [60, 61]. Hiramoto et al. reported the effects of ARs on denervation-induced muscle atrophy studied in mouse models [59]. According to this investigation, ARs supplement prevented the reduction of myofibers and their mass in skeletal muscle caused by denervation, although ARs failed to suppress the upregulated gene expression of muscle atrophy associated ubiquitin ligases and autophagy-associated genes. The expression of p62, considered as a marker of autophagy, was thus higher in the atrophied muscle of mice fed with ARs-supplemented diet. Based on these results, the authors proposed that energy metabolism in the atrophic muscle of mice provided with ARs was altered from the oxidation of glucose to the oxidation of fatty acids instead, which prevented the infiltration of muscle tissue with fat and protected it from autophagy [59].

### 5.7. ARs Exhibit Neuroprotective Potential

Gradual reduction of cell number in certain populations of neurons in the central nervous system results in the development of neurodegenerative disorders (ND) [62], which mostly affect elderly people and manifest in cognition or memory impairments leading to the individual's death [63]. In the light of this, the search for efficient neuroprotective agents is an issue of the day. Phenolic compounds, considered as multitarget drugs, are promising factors able to prevent oxidative stress and modulate the activity of endogenous enzymes and receptors associated with neuroprotection. As it was shown recently, wheat bran-originated ARs demonstrated neuroprotective effects in H2O2-treated HT22 cells through acting as antioxidants and controlling the Nrf2-ARE pathway [64]. The latter is known to play a crucial role in cellular protection upon exposure to oxidative stress. The mouse immortalized neuronal HT22 cells derived from the hippocampus, one of the key brain structures in charge of memory formation [65], are frequently used as an *in vitro* model to investigate the mechanisms of neuronal cell death and neurodegenerative diseases [66]. Oxidative stress in such models is stimulated by H_2_O_2_, which serves as a source of reactive oxygen species (ROS), thus demonstrating neurotoxicity properties and dramatically decreasing cell viability [67]. In the study of Fan et al., the HT22 cells pretreated with ARs before H_2_O_2_ treatment demonstrated a significant dose-dependent increment in viability and ROS decline, indicating that ARs could protect cells from H2O2-induced oxidative stress [64]. Moreover, the oxidative damage of cells was inhibited by ARs through the activation of the Nrf2/ARE signaling pathway, for the ARs are believed to promote the translocation of Nrf2 to the nucleus and to upregulate its transcription. Following this scenario, the activated nuclear form of Nrf2 is likely to further induce phase II antioxidant enzyme gene transcription [68]. These suggestions explain the marked increase in the expression of HO-1, NQO1, GCLC, and GCLM in HT22 cells in response to ARs. Taken together, these data imply that ARs could be used as an excellent dietary component with the neurodegenerative protective effect to improve human health.

### 5.8. Anticancer Properties of ARs

Numerous epidemiological studies report the anticancer effect of a whole grain diet against breast, colon, and prostate cancer [25, 69] and, in particular, the ability of wheat bran-originated ARs to block the proliferation of human prostate adenocarcinoma cells and colon cancer cells [11, 69]. The antitumor mechanism of ARs consists in the destruction of tumor cells' DNA and the prevention of DNA repair in them [70], thus increasing the rate of cell death with genetic toxicity [71], which stops the formation of new cancer cells. As recent investigations indicate, natural plant extracts such as flavonoids and polyphenols are able to stimulate apoptosis- and autophagy-associated intracellular pathways to precipitate the death of tumor cells and to stop their proliferation [69].

In the study of Oskarsson and Ohlsson [72] carried out on the human adrenocortical cell line H295R, the nontoxic concentrations of C15 : 0 and C19 : 0 ARs directly affected the steroidogenesis; namely, they reduced the synthesis of testosterone and decreased the secretion of estradiol, cortisol, and aldosterone. The authors proposed that the decline in the secretion of the hormones could appear due to the inhibition of the activity of CYP17 enzyme, which is in charge of the conversion of pregnenolone to 17*α*-OH-pregnenolone and of progesterone to 17a-OH-progesterone. The blocked enzyme's activity might result in the shift from glucocorticoid and androgen synthesis toward the synthesis of aldosterone observed in the study. This hypothesis is supported by the fact that, under the treatment of cells with mixtures of ARs with lower concentrations, the decrease in androgen and cortisol secretion accompanied a slight increase in aldosterone production. The results are of particular importance, as CYP17 is considered as a key enzyme in the synthesis of steroid hormones and a key target in the treatment of prostate cancer [73], in which cells depend on androgens needed for their growth. Thus, the inhibition of CYP17 activity by ARs blocking androgen production may be a promising approach in anticancer therapy to reduce the proliferation of prostate cancer cells. Other studies also confirm the association between rye intake or ARs consumption and the probability of oncological disease development. For instance, according to Torfadottir et al. [74], adults who include rye bread in their daily rations have a reduced risk of prostate cancer, particularly, advanced prostate cancer, development. On the other hand, in another investigation they reported no significant protective effects of whole grain consumption and the associated AR metabolites in plasma on incident prostate cancer [75].

Similarly to the correlation between the level of androgens and the risk of prostate cancer, prolonged exposure to estrogens may lead to the development of breast cancer, and more than 60% of breast cancers are found to be estrogen-dependent [76]. As ARs were shown to decline estradiol secretion, rye product consumption may be recommended as a countermeasure capable of reducing the risk of breast cancer development [77]. Along with ARs leading to decreased plasma estrogen levels, the generally high dietary fiber intake was reported to be associated with the lower risk of breast cancer as well [78, 79]. Besides the mechanism described earlier, ARs, as highly lipophilic molecules, are proposed to affect steroid hormone production through the accumulation in tissues enriched in lipids, such as adipose tissue, testis, ovaries, and adrenal cortex, which are in charge of steroidogenesis [72].

ARs reliably not only reduces the risk of prostate cancer in men and breast cancer in women, but is also associated with the decreased risk of colorectal cancer development in both genders [80, 81]. Kyro et al. in their study of ARs intake association with colorectal cancer, conducted on a large, multicenter cohort with more than one million individuals from 10 European countries, considered the plasma concentrations of different ARs homologs, namely, C17 : 0, C19 : 0, C21 : 0, C23 : 0, and C25 : 0, and the incidence rate ratio (IRR) of distal colon cancer in matched case-control pairs [80]. In participants with ARs blood concentrations above 80 nM (>99 nM in men and >84 nM), the IRR of distal colon cancer was reduced by 52% (78 cases, 114 controls). Moreover, a 17% reduction in IRR of colon cancer was reported for Scandinavian participants (252 case-control pairs). The authors thus indicate an inverse correlation between the total concentrations of ARs in plasma and the risk of colon and distal colon cancer development, although the phenomenon was observed only in those individuals who come from areas with high, stable, and frequent ARs intake, namely, Central Europe and Scandinavia.

Another study of the AR-colorectal cancer relationship, conducted with the participation of approximately one hundred twenty thousand men and women aged 30–64 years, reported a 64% decreased risk of distal colon cancer (198 cases-control pairs) in individuals with higher ARs plasma concentrations (>118.6 nM for men and >91.7 nM for women) compared to those with lower ARs levels in blood (≤35.3 nM for men and <27.5 nM for women) [81].

Anticancer properties of ARs are being characterized in multiple *in vitro* studies using human hepatocarcinoma, ovarian, cervical, colon, lung, central nervous system, and breast cancer cell lines as models, and some of the results to date are perfectly observed in the review of Kruk et al. [1]. In brief, ARs are able to inhibit the growth of cancer cells in the human breast, lung, and central nervous system. In the study of Sanchez et al. [82], they tested the cytotoxicity of five isolated natural ARs on MCF-7, H-460, and SF-268 cell lines and demonstrated the 800% stronger inhibitory effect of ARs over the commonly known cytostatic agent Adriamycin. The results of this study are in concordance with the previously reported antiproliferative effect of 14 different ARs isolated from the leaves of a tropical ornamental tree applied to human breast carcinoma, lung carcinoma, and central nervous system carcinoma cells [83].

Two human hepatocarcinoma cell lines, HepG2 and Hep3B, were used to demonstrate the cytotoxic effect of 5-alkylresorcinol (5-AR) from the leaves of *L. molleoides*, and in both of them 24-hour exposure to 5-AR resulted in the fragmentation of DNA and the nuclear condensation, characteristic for apoptotic cells [84]. Interestingly, the cell death induction in the cultures occurred in a p53-independent way. Other investigations of the last three decades confirm the property of ARs to inhibit the growth of tumor cells, particularly, ovarian cancer cells [85], human prostate adenocarcinoma (PC3) cells [69], breast cancer cell line [82, 83, 86], and cervical cancer (HeLa) cell line [87]. Taken together, these facts propose that ARs can be promising adjuvants in anticancer therapy.

The exact mechanisms of ARs anticancer properties remain unclear due to multiple ARs targets and the complexity of interrelations between various biochemical actions. Apparently, ARs affect cellular morphology via the fragmentation of DNA and the condensation of nuclei leading to apoptosis [84], in particular, in cells already damaged by genotoxic agents [71]. At the same time, other researchers report the antioxidant properties of ARs, which manifest in the reduction of the amount of ROS and/or in the inhibition of the enzymes involved in the production of free radicals under physiological conditions [88]. Although the anticancer properties of ARs are obvious, further studies are needed to elucidate the exact molecular mechanisms of ARs action.

### 5.9. ARs and Metabolic Health

It is generally accepted that Mediterranean diet, which consists of a lot of fruits, vegetables, legumes, nuts, and whole grain products, is beneficial for human metabolic health. The advantages of such a diet are not only in getting an appropriate amount of vitamins, microelements, and antioxidant agents, but also in the consumption of such regulatory substances like ARs. As it was shown by Rejman et al. on 3T3-L1 cell culture, ARs prevented the cytoplasmic accumulation of triglycerides, possibly by the *in vivo* sirtuin-dependent inhibition of triglyceride synthesis [89]. Negative correlation between the level of alkylresorcinols and nonesterified fatty acids concentrations in human plasma samples was demonstrated after the switch to a whole grain diet [90]. Additionally, ARs were shown to inhibit lipolysis carried out by hormone-sensitive lipase [91] and to block the activity of glycerol-3-phosphate dehydrogenase, which is in charge of triglyceride accumulation [52]. The addition of solely ARs to the «high fat/high sucrose (FS) diet» managed not only to suppress the accumulation of triglycerides in the liver of mice, but also to overcome hyperinsulinemia and hyperleptinemia in the FS group of animals [46]. Other stunning results of this experiment include the marked ARs-dependent reduction of fasting blood glucose concentrations and the suppression of both glucose intolerance and insulin resistance induced by the FS diet. Oishi et al. also found that ARs significantly enhanced insulin-stimulated hepatic Akt phosphorylation, which might be associated with the increased *irs* gene expression in the liver. Besides, circulating plasma total cholesterol level decreased along with the increased fecal cholesterol excretion, which clearly contributes to the ARs-mediated suppression of the accumulation of lipids in the liver as well as the overcoming of glucose intolerance and insulin resistance. Thus, ARs successfully prevented diet-induced obesity in mice, and it is supported by the fact that, despite the similar daily caloric intake, mice fed with FS diet gained more weight compared to the group provided with ARs along with the FS ration. According to the study, ARs significantly suppressed the FS-induced increase in plasma leptin concentration and concomitant adipose mRNA expression. Since leptin is known as one of the major satiety signaling molecules, which travel from adipose tissue to the hypothalamic centers that regulate appetite [92], ARs might suppress leptin resistance in the hypothalamus. However, according to the investigation of Oishi et al., ARs did not affect either gluconeogenesis in the liver, or the expression of genes associated with gluconeogenesis, such asG6pc and Pck1, but significantly enhanced the expression of *Irs1* mRNA during the feeding period compared to the FS diet, and this corresponded to Akt phosphorylation [46]. Undoubtedly, deeper studies are required to reveal the underlying mechanisms of the AR-induced increase in hepatic insulin sensitivity.

The results of the study described above are well consistent with the research of Malin et al., in which they showed the significant improvement in the glucose-stimulated insulin secretion (GSIS) response in middle-aged overweight/obese adults at risk for type 2 diabetes, who followed a whole grain diet compared to those provided with refined grains [93]. Thus, the inclusion of whole grains in a daily ration may help an individual in the compensation for insulin resistance due to *β*-cell stimulation and additional activation of insulin secretion. Such an approach could serve to prevent the progression to diabetes in an at-risk obese population with prediabetes. The authors suggested several mechanisms by which whole grains may increase GSIS. First, the diet rich in whole grains promotes weight loss due to the reduction of fat mass [94], which, in its turn, can lower the blood concentrations of lipids and/or inflammatory factors known to disturb *β*-cell insulin secretion [95]). Second, gut hormones from the family of incretins, such as GLP-1 (glucagon-like peptide 1) and GIP (gastric inhibitory polypeptide), which are secreted in response to nutrient consumption [96], are hypothesized to be elevated together with insulin in the individuals provided with whole grain products. However, according to the observations of Malin et al., whole grain intake had no effect on gut hormones compared to refined grains, and both whole grain and refined grain interventions reduced body weight and fat loss [93].

Diet enriched in whole grains is associated with lower concentrations of serotonin, taurine, and glycerophosphocholine in blood [97]. Martin at al. demonstrated the decreased colonic serotonin synthesis in mice fed with rye bran compared to those supported with cellulose as dietary fiber [98]. Although gut-derived serotonin is an important mediator in normal gastrointestinal physiology, the inhibition of its synthesis is evidenced to reduce obesity and metabolic dysfunction and ameliorate type 2 diabetes [99]. Since elevated blood serotonin is linked to the reduced glycemic control, the findings of the whole grain-induced decrease in serotonin production are intriguing. At the same time, the influence of ARs on the synthesis of serotonin in the intestine, as well as their role in the improvement of sensitivity to insulin and the reduction of the risk of type 2 diabetes development, remains to be elucidated.

As it was mentioned previously, ARs consumption may decrease body weight by inhibiting nutrient absorption. ARs contain hydrophobic alkyl chains able to react with human digestive enzymes such as *α*-glucosidase, trypsin, and aldose reductase, thus affecting carbohydrate digestion [22]. Additionally, Song et al. in their research of *α*-glucosidase kinetics managed to demonstrate the 4-hexylresorcinol potential as a noncompetitive reversible inhibitor of the enzyme. The substance tested also presented the ability to interfere with nonenzymatic glycation reactions, thus reducing the formation of fructosamines and blocking the synthesis of *α*-dicarbonyl compounds and advanced glycation end products (AGEs) [100]. *In vitro* investigations confirmed ARs inhibitory effects on *α*-glucosidase [101]. Based on these data, 4-hexylresorcinol can be considered as a possible preventive and therapeutic agent for adult-onset diabetes.

Another beneficial effect of a whole grain diet is a lower risk of fatty liver, although the underlying mechanisms remain unknown [102]. Nonalcoholic hepatosteatosis is closely associated with insulin resistance and the promotion of impaired glucose tolerance [103]. *In vivo* studies in mice demonstrated the significant ARs-dependent suppression of hepatic triglyceride accumulation induced by fat-rich diet, although neither the concentrations of circulating free fatty acids, nor the mRNA expression of both lipogenic- and *β*-oxidation-related genes were affected [46]. Besides, ARs were shown to increase the fecal excretion of total lipids and cholesterol and to significantly reduce the amounts of fecal bile acids, although the effect was strong only in conditions of elevated hepatic bile secretion. These findings suggest that the increased fecal cholesterol excretion was independent of the increased excretion of fecal bile acids and most likely linked to the ability of ARs as hydrophobic compounds, to reduce the capacity to carry micellar lipids by binding bile acids and suppressing intestinal cholesterol absorption, although the precise mechanism remains to be clarified [46].

## 6. Conclusions

**Figure 5:**
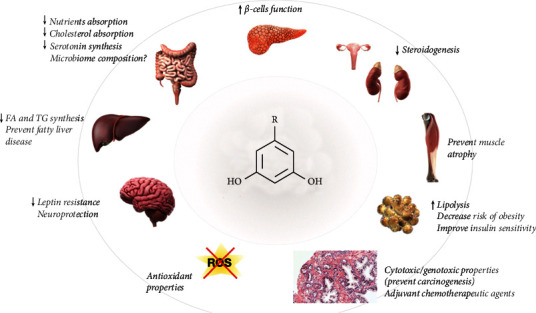
The main regulatory functions of ARs according to *in vivo* and *in vitro* studies.

This systematic review is aimed at the summary of the beneficial effects of ARs on human health. Plenty of evidence indicating diverse ARs bioactivities has been accumulated in recent years (Figure 5).

*In vivo* and *in vitro* studies, as well as epidemiological investigations, have shown that ARs can affect many physiological and pathological processes related to the immune system, metabolic regulation, cell signaling, and gene expression regulation. Currently, ARs are used as biomarkers of whole grain wheat and rye product consumption and are proposed to be utilized as adjuvant anticancer and antimicrobial therapeutic agents. Due to ARs ability to take on the role of autoregulators of microbial activity, further investigations concerning their role in «gut microbiome-host organism» interactions are needed.

There are some observations concerning ARs neuroprotective, muscle-protective, and metabolic improving effects. Strong evidence was received about the positive influence of a whole grain diet and increased levels of ARs on human metabolic health.

High plasma concentrations of ARs are proved to be associated with the reduced risk of colon, prostate, and breast cancers. Numerous *in vitro* studies report the highly cytotoxic properties of high concentrations of ARs for certain types of cancer, particularly, human colon, breast, lung, central nervous, ovarian, cervical, and prostate tumors, and hepatocarcinoma cancer cell lines. Furthermore, the phenolic ring and alkyl chain as structural components of ARs are important for the inhibition of human cancer cell proliferation, although the exact mechanisms of the toxicity are not clear to date.

There are some hypothesized molecular mechanisms of ARs action as bioregulators, including their ability to affect all enzyme-regulated processes in cells, suppress lipolysis in adipocytes, cause genotoxicity, insert into the membranes of erythrocytes, and exert indirect antioxidant capacity.

The characterization of ARs properties provides further support for public health recommendations emphasizing diets rich in whole grains as potentially preventive against metabolic, immune, oncological, and other chronic diseases.
